# Identification of the Complete Chloroplast Genome of *Malus zhaojiaoensis* Jiang and Its Comparison and Evolutionary Analysis with Other *Malus* Species

**DOI:** 10.3390/genes13040560

**Published:** 2022-03-22

**Authors:** Xun Wang, Daru Wang, Ning Gao, Yuepeng Han, Xiaofei Wang, Xiang Shen, Chunxiang You

**Affiliations:** 1 State Key Laboratory of Crop Biology, College of Horticultural Science and Engineering, Shandong Agricultural University, Taian 271018, China; wx20145015@126.com (X.W.); 15953327292@163.com (D.W.); xfwang2004@163.com (X.W.); 2 Institute of Advanced Agricultural Sciences, Peking University, Weifang 261325, China; 18754445662@163.com; 3 Key Laboratory of Plant Germplasm Enhancement and Specialty Agriculture, Wuhan Botanical Garden, Chinese Academy of Sciences, Wuhan 430074, China; yphan@wbgcas.cn; 4 National Research Center for Apple Engineering and Technology, Shandong Collaborative Innovation Center for Fruit and Vegetable Production with High Quality and Efficiency, Shandong Agricultural University, Taian 271018, China

**Keywords:** *Malus zhaojaoensis*, chloroplast genome, repeat sequences, codon usage, phylogenetic tree

## Abstract

The genus *Malus* is rich in species and many of its plastid genomes have been released. However, limited resources and few markers are not conducive to the comparison of differences among species and resource identification and evaluation. In this study, the complete chloroplast genome of *Malus zhaojiaoensis* was studied by NGS sequencing, with a total length of 159998 bp. It consists of four regions, LSC (88,070 bp), IRB (26,359 bp), SSC (19,210 bp) and IRA (26,359 bp). *M. zhaojiaoensis* cp genome contained a total of 111 genes made up of three classes: 78 coding sequences, 29 tRNA genes, and four rRNA genes. In addition, a total of 91 SSRs and 43 INEs were found in the *M. zhaojiaoensis* cp genome, which was slightly different from *M. baccata* and *M. hupehensis* in number. The analysis of codon usage and RNA editing showed that high-frequency codons tended to end at A/U bases and RNA editing mainly occurred at the second codon. Comparative genome analysis suggested that the cp genomes of eight *Malus* species had higher overall similarity, but there were more variation hotspots (*rps16_trnK-UUU*, *trnG-UCC_atpA*, *atpH_atpF*, *trnT-GGU_psbD*, etc.) in the LSC region. By building evolutionary trees, it can be clearly observed that *M. zhaojiaoensis* formed a large group with eight species of *Malus*, but was relatively independent in differentiation. In conclusion, this study provides high-quality chloroplast genome resources of *M. zhaojiaoensis* and discusses the genetic variation characteristics of *Malus* genus. The findings of this study will provide a good reference for plastid genome assembly and interspecific comparison in the future.

## 1. Introduction

The genus *Malus* is rich in species and widely distributed and cultivated throughout the world [1]. According to the principle of ecological geography, *Malus* genus can be divided into two groups: cultivated species and wild species [2]. Among them, cultivated species are greatly affected by human activities and are mainly used for fresh food and processing. Of course, some species are also used as ornamental plants [3]. Wild species exist in natural distribution areas and are often used as rootstock resources and breeding materials in production due to their good adaptability of introduction and affinity for grafting [4]. As a wild species of *Malus* native to China and widely distributed in northeast, northern and southwest China, *M. baccata* (L.) Borkh. has diverse populations and variation types [5], which is of great value in producing activity and scientific research. *M. zhaojiaoensis* Jiang is a new species of *Malus* found in Zhaojue Xian, Sichuan Province [6]. According to the report [6], *M. zhaojiaoensis* has oval leaves, reddish flowers, and small fruits, which are of great ornamental value. It has similar characteristics with *M. baccata* and *M. rockii* [7], but its evolutionary status and genetic relationship is still ambiguous and needs further research.

There are many methods to study plant genetic evolution, including morphological comparison, biochemical methods (isozymes, etc.) and a wide range of molecular markers (Restriction Fragment Length Polymorphism, Amplified Fragment Length Polymorphism, Random Amplified Polymorphism DNA, Simple Sequence Repeat, Single Nucleotide Polymorphism) [8]. In recent years, with the emergence of high-throughput sequencing technology and the development of bioinformatics based on multiple omics, methods such as resequencing, comparative genomics, and chloroplast genome assembly provide new and efficient solutions for species classification and variation detection [9]. Chloroplast DNA exists in plant chloroplasts in a double chain ring shape, and most plant chloroplast genomes consist of four typical parts, namely a large single copy region (LSC), a small single copy region (SSC), and two inverted repeat regions (IRA and IRB) [10]. Compared with nuclear genomes, chloroplast genomes are relatively conservative and small, so they play an irreplaceable role in species identification [11].

Up to now, there have been numerous studies on chloroplast genomes of *Malus* plants, such as *M. toringoides* [12], *M. kansuensis* [13], *M. sieboldii* [14], *M. sylvestris* [15], *M. hupehensis* [16], and *M. prattii* [17]. This has played a positive role in the utilization and protection of *Malus* resources. In this paper, the complete chloroplast genome of *M. zhaojiaoensis* was assembled based on second-generation sequencing data and bioinformatics methods, and its basic characteristics, sequence similarity, and evolutionary relationship were analyzed and compared. The above results can provide reference for explaining the genetic variation characteristics of *M. zhaojiaoensis* and *Malus*. At the same time, the findings of this paper will play a positive role in the conservation and development of wild species such as *M. zhaojiaoensis*.

## 2. Materials and Methods

### 2.1. Sample Collection and DNA Sequencing

Young, fresh and clean leaves of *M. zhaojiaoensis* Jiang tree were collected from Fruit Research Institute, Chinese Academy of Agricultural Sciences (Xingcheng city, Liaoning Province, China). Total DNA was extracted by improved CTAB (Cetyltrimethylammonium Bromide) method [18]. Construction of genomic DNA library and DNA sequencing were performed using Illumina HiSeq X Ten system [19]. 150 bp paired-end sequences were sequenced in Illumina platform, and insert size was 350 bp. Adapter sequences were removed by Trimmomatic v0.39 software and adapter-trimmed raw reads were used for subsequent assembly analysis [20].

### 2.2. Chloroplast Genome Assembly and Annotation

GetOrganelle v1.7.5.0 [21] and NOVOplasty4.2 [22] were used for chloroplast genome assembly. In GetOrganelle, the values of kmers were set to 21, 45, 65, 85, 105. The complete chloroplast genome sequence of *M. zhaojiaoensis* was obtained after the assembly results were confirmed. After that, the sequence was submitted to PGA (Plastid Genome Annotator) program [23] for gene annotation, and the annotation information of CPGAVAS [24] and GeSeq [25] was also used for integration and merger. Chloroplast genome sequence and annotation file were manually revised and submitted to NCBI website (https://www.ncbi.nlm.nih.gov/, accessed on 20 February 2022). Its accession number was OM793283. OrganellarGenomeDRAW [26] was used to graphically display the annotations of cp genome, and different genes were distinguished by different colors.

### 2.3. Description of Basic Characteristics of Chloroplast Genome

The basic characteristics of chloroplast genome include size and structure, GC content, gene classification and repeat sequence, and so on. The above analysis was accomplished by Python (https://www.python.org/, accessed on 10 February 2022), Geneious (https://www.geneious.com/, accessed on 15 February 2022), MISA (https://webblast.ipk-gatersleben.de/misa/, accessed on 8 February 2022) [27], Vmatch (http://vmatch.de/, accessed on 8 February 2022), etc. Regarding search parameters of simple sequence repeats (SSRs), the minimum number of repetitions was set to 10 (mononucleotide), 5 (dinucleotide), 4 (trinucleotide), 3 (tetranucleotide), 3 (pentanucleotide), and 3 (hexanucleotide). In the identification of interspersed nuclear elements (INEs), the parameters were as follows: Minimal Repeat Size (30) and Hamming Distance (3). The cp genomes of *M. baccata* (MK896774) and *M. hupehensis* (MK020147) were used for comparative analysis.

### 2.4. Analysis of Codon Usage Characteristics and RNA Editing Sites

All the coding sequences were extracted based on the chloroplast genome annotation of *M. zhaojiaoensis*. The calculation and comparison of RSCU (relative synonymous codon usage) value was operated in CodonW v1.4.2 (http://codonw.sourceforge.net/, accessed on 12 February 2022) programme. PREP-Cp (http://prep.unl.edu/, accessed on 16 February 2022) was used to predict potential RNA editing sites in coding sequences [28].

### 2.5. Chloroplast Genome Alignment, Boundary and Evolutionary Analysis

Several chloroplast genomes of *Malus* and other genera were searched from NCBI nucleotide database, including *M. hupehensis* (MK020147), *M. baccata* (MK896774), *M. halliana* (MT246302), *M. micromalus* (MF062434), *M. prattii* (MH929090), *M. prunifolia* (KU851961), *M. sieboldii* (MT593044), *M. toringoides* (MT483999), *M. yunnanensis* (MH394387) and *Crataegus hupehensis* (*Crataegus*, MW201730), etc. The homology and similarity of the cp genomes of four *Malus* genera were compared in Circoletto (http://tools.bat.infspire.org/circoletto/, accessed on 18 February 2022) [29]. The sequences of chloroplast genomes were aligned in VISTA (https://genome.lbl.gov/vista/index.shtml, accessed on 16 February 2022); Shuffle-LAGAN was designated as alignment program [30]. IRscope was used to visualize the junction sites of the chloroplast genomes [31]. Phylogenetic and cluster analysis was performed in HomBlocks (https://github.com/fenghen360/HomBlocks, accessed on 18 February 2022) [32] and MEGA tool (https://www.megasoftware.net/, accessed on 18 February 2022) [33], and *C. hupehensis* (*Crataegus*) was selected as outgroup. Two types of evolutionary trees (Neighbour-Joining tree and Maximum Likelihood tree) were constructed in MEGA X, both of which were examined with bootstrap method (1000 replications). In addition, ClustalW (Codons) alignment method and Jukes-Cantor substitution model were selected for NJ evolutionary trees based on single-copy genes (*matK* and *rbcL*).

## 3. Results

### 3.1. Characteristics of the Chloroplast Genome of M. zhaojiaoensis

#### 3.1.1. Chloroplast Genome Map and Structure

**Figure 1:**
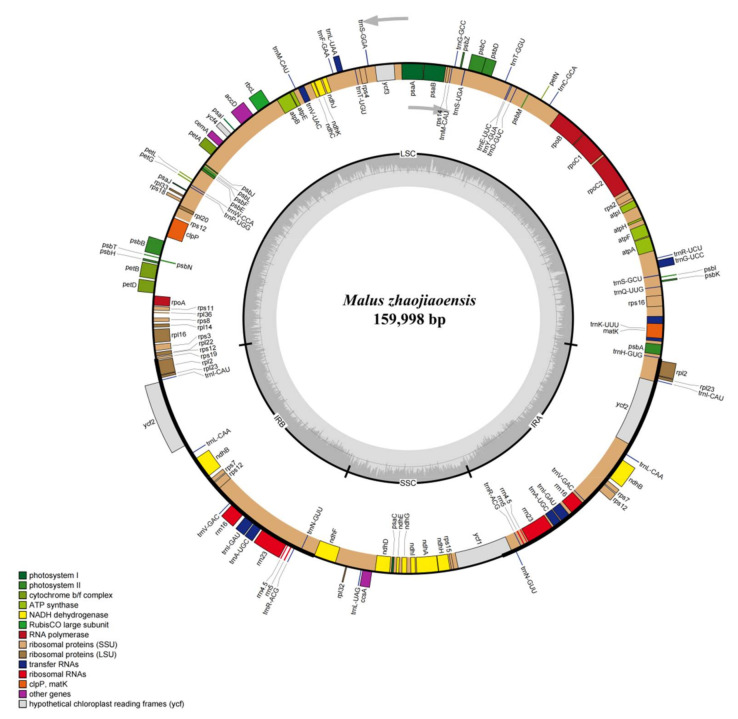
The map of complete chloroplast genome in *M. zhaojiaoensis*. Different colored blocks on the outer ring represent different genes, while the shadow on the inner ring indicates GC content.

After genome sequencing and assembly, a complete chloroplast genome sequence of *M. zhaojiaoensis* was generated, with a total length of 159,998 bp and an overall GC content of 36.6%. Like other chloroplasts, the chloroplast genome of *M. zhaojiaoensis* has a double chain ring structure and includes four distinct and typical regions (two single copy regions and two inverted repeat regions). The sequence lengths of the tetrads were 88,070 bp (LSC: 1–88,070), 26,359 bp (IRB: 88,071–114,429), 19,210 bp (SSC: 114,430–133,639) and 26,359 bp (IRA: 133,640–159,998), respectively (Figure 1). By statistical calculation, GC content in IR regions (42.7%) was higher than that in the whole cp genome (36.6%) of *M. zhaojiaoensis*, indicating that the two inverted repeat regions (IRA and IRB) were relatively stable.

#### 3.1.2. Gene Annotation and Classification of Chloroplast Genome

Genetic annotations were completed on the assembled genome of *M. zhaojiaoensis*, which contains a total of 111 genes made up of three classes: CDS (coding sequence, 78), tRNA (transfer RNA, 29) and rRNA (ribosomal RNA, 4). According to function, all annotated genes could be divided into four main categories, photosynthesis, self-replication, others, and unknown (Table 1). Six *atp* (subunits of ATP synthase), 15 *psb* (subunits of photosystem II), 11 *ndh* (subunits of NADH-dehydrogenase), 6 *pet* (subunits of cytochrome b/f complex), 5 *psa* (subunits of photosystem I) and 1 *rbc* (subunit of rubisco) genes were involved in photosynthesis. Nine *rpl* (large subunit of ribosome), 4 *rpo* (DNA dependent RNA polymerase), 12 *rps* (small subunit of ribosome), 4 *rrn* (rRNA), and 29 *trn* (tRNA) genes were associated to self-replication. In addition to the above two parts, there were nine genes belonging to other and unknown classes, namely *accD*, *ccsA*, *cemA*, *clpP*, *matK* and *ycf* (*ycf1*, *ycf2*, *ycf3*, *ycf4*).

Among 111 genes, six CDS (*ndhB*, *rpl2*, *rpl23*, *rps7*, *rps12*, *ycf2*), eight tRNA (*trnA-UGC*, *trnI-CAU*, *trnI-GAU*, *trnL-CAA*, *trnM-CAU*, *trnN-GUU*, *trnR-ACG*, *trnV-GAC*), four rRNA (*rrn4.5*, *rrn5*, *rrn16*, *rrn23*) genes had two copies, and the rest had only one copy. Due to the existence of duplicate genes, the actual number of genes in the chloroplast genome of *M. zhaojiaoensis* reached 129, including 84 encoding genes, 37 tRNA and 8 rRNA genes.

**Table 1:** Gene classification of chloroplast genome in *M. zhaojiaoensis*.

Gene Function	Gene Group	Code	Gene Number	Gene Name
Photosynthesis	Subunits of ATP synthase	*atp*	6 (6)	*atpA*, *atpB*, *atpE*, *atpF*, *atpH*, *atpI*
	Subunits of photosystem II	*psb*	15 (15)	*psbA*, *psbB*, *psbC*, *psbD*, *psbE*, *psbF*, *psbH*, *psbI*, *psbJ*, *psbK*, *psbL*, *psbM*, *psbN*, *psbT*, *psbZ*
	Subunits of NADH-dehydrogenase	*ndh*	11 (12)	*ndhA*, *ndhB (×2)*, *ndhC*, *ndhD*, *ndhE*, *ndhF*, *ndhG*, *ndhH*, *ndhI*, *ndhJ*, *ndhK*
	Subunits of cytochrome b/f complex	*pet*	6 (6)	*petA*, *petB*, *petD*, *petG*, *petL*, *petN*
	Subunits of photosystem I	*psa*	5 (5)	*psaA*, *psaB*, *psaC*, *psaI*, *psaJ*
	Subunit of rubisco	*rbc*	1 (1)	*rbcL*
Self replication	Large subunit of ribosome	*rpl*	9 (11)	*rpl14*, *rpl16*, *rpl2 (×2)*, *rpl20*, *rpl22*, *rpl23 (×2)*, *rpl32*, *rpl33*, *rpl36*
	DNA dependent RNA polymerase	*rpo*	4 (4)	*rpoA*, *rpoB*, *rpoC1*, *rpoC2*
	Small subunit of ribosome	*rps*	12 (14)	*rps11*, *rps12 (×2)*, *rps14*, *rps15*, *rps16*, *rps18*, *rps19*, *rps2*, *rps3*, *rps4*, *rps7 (×2)*, *rps8*
	rRNA	*rrn*	4 (8)	*rrn4.5 (×2)*, *rrn5 (×2)*, *rrn16 (×2)*, *rrn23 (×2)*
	tRNA	*trn*	29 (37)	*trnA-UGC (×2)*, *trnC-GCA*, *trnD-GUC*, *trnE-UUC*, *trnF-GAA*, *trnG-GCC*, *trnG-UCC*, *trnH-GUG*, *trnI-CAU (×2)*, *trnI-GAU (×2)*, *trnK-UUU*, *trnL-CAA (×2)*, *trnL-UAA*, *trnL-UAG*, *trnM-CAU (×2)*, *trnN-GUU (×2)*, *trnP-UGG*, *trnQ-UUG*, *trnR-ACG (×2)*, *trnR-UCU*, *trnS-GCU*, *trnS-GGA*, *trnS-UGA*, *trnT-GGU*, *trnT-UGU*, *trnV-GAC (×2)*, *trnV-UAC*, *trnW-CCA*, *trnY-GUA*
Others	Subunit of Acetyl-CoA-carboxylase	*acc*	1 (1)	*accD*
	c-type cytochrom synthesis gene	*ccs*	1 (1)	*ccsA*
	Envelop membrane protein	*cem*	1 (1)	*cemA*
	Protease	*clp*	1 (1)	*clpP*
	Maturase	*mat*	1 (1)	*matK*
Unknown	Conserved open reading frames	*ycf*	4 (5)	*ycf1*, *ycf2 (×2)*, *ycf3*, *ycf4*

The number in parentheses in the fourth column is the total number of duplicates contained, and the fifth column (×2) refers to two copies of the gene.

In the chloroplast genome of *M. zhaojiaoensis*, most genes do not have introns, but there are still some genes that contain one to two introns (Table 2). The total number of genes with introns was 23 (15 CDS and 8 tRNA genes), of which 19 had one intron, and four (*ycf3*, *rps12*, *rps12*, *clpP*) had two introns. In terms of genome location and distribution, 12 (eight CDS and four tRNA genes) of the 23 genes were located in LSC region of cp genome, accounting for the largest proportion. Four genes (*rpl2*, *ndhB*, *trnI-GAU*, *trnA-UGC*) were located in IRA and IRB, and only one gene (*ndhA*) was distributed in SSC region. In addition, it is worth noting that the *rps12* gene encoding ribosomal subunit S12 protein consists of two distant parts of the cp genome, one in the LSC region and the other in the IR region, and it undergoes special trans-splicing during transcription.

### 3.2. The Type and Distribution of Repeat Sequences

#### 3.2.1. Simple Sequence Repeats

**Figure 2:**
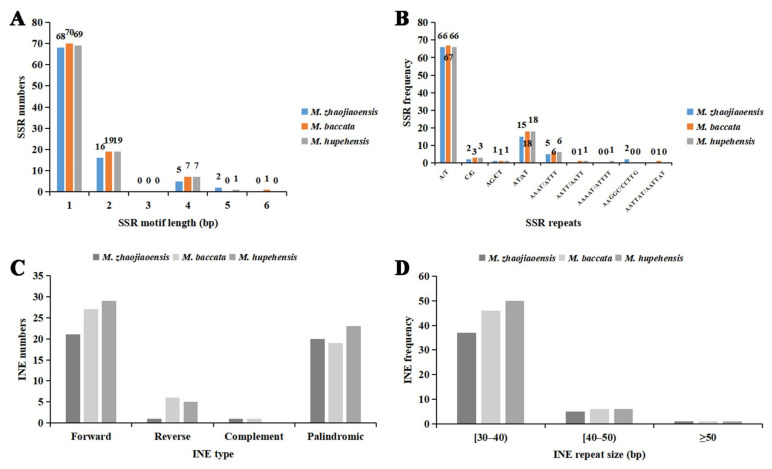
The comparison of SSRs and INEs of chloroplast genomes in three *Malus* species. (**A**): Numbers of SSRs with different length; (**B**): Frequency of different types of SSRs; (**C**): Numbers of different types of INEs; (**D**): Frequency of INEs with different length.

Six types (mono-, di-, tri-, tetra-, penta-, and hexa nucleotide) of SSRs were identified in the MISA-web. In chloroplast genome of *M. zhaojiaoensis*, single nucleotide repeat (68) was the most frequent SSR (Figure 2A, Table S1), followed by dinucleotide repeat (16). In addition, there were five tetranucleotide SSR markers and two pentanucleotide SSR markers. No trinucleotide repeats and hexanucleotide repeats were found in chloroplast genome of *M. zhaojiaoensis*. For comparison, SSR types and numbers in *M. baccata* and *M. hupehensis* cp genome were also identified and counted in this study. The total number of SSRs in the three cp genomes were similar, 91 in *M. zhaojiaoensis*, 97 in *M. baccata* and 96 in *M. hupehensis*. Unlike *M. zhaojiaoensis* and *M. hupehensis*, there was no pentanucleotide repeat type in *M. baccata*, but the *M. baccata* cp genome had one hexanucleotide SSR (Figure 2A).

For mononucleotide SSRs, A/T repeat was most abundant with 66, 67 and 66 times in *M. zhaojiaoensis*, *M. baccata* and *M. hupehensis*, respectively. It is noteworthy that the composition of pentanucleotide repeats in *M. zhaojiaoensis* was AAGGC/CCTTG, and that in *M. hupehensis* was AAAAT/ATTTT (Figure 2B). The only hexanucleotide repetition in *M. baccata* was the ATAATT unit repeated three times, with a total base length of 18 bp.

#### 3.2.2. Interspersed Nuclear Elements

Interspersed repeats are another important repeating element in the genome and there are abundant INEs (interspersed nuclear elements) in the chloroplast genome of plants. Four types (forward_direct, reverse, complement, palindromic) of INEs were calculated in REPuter. In the cp genome of *M. zhaojiaoensis*, the INEs contained 21 forward repeats, 20 palindromic repeats, 1 reverse repeat, and 1 complement repeat (Figure 2C, Table S2). The *M. baccata* cp genome had 27 F, 19 P, 6 R, and 1 C type repeats, while there were 29 forward, 23 palindromic, 5 reverse, and 0 complement repeats in *M. hupehensis* (Figure 2C). In general, the number of INEs in *M. zhaojiaoensis* (43) was significantly less than that in *M. baccata* and *M. hupehensis*, and the total number of INEs in *M. baccata* (53) and *M. hupehensis* (57) was close.

In addition, from the perspective of repeated fragment size, it can be found that most of the sequence lengths were concentrated in the range of 30–50 bp, especially 30–40 bp. There was one INE repeat with a sequence length of more than 50 bp in each of the chloroplast genomes of three *Malus* species (Figure 2D).

### 3.3. Relative Synonymous Codon Usage

**Figure 3:**
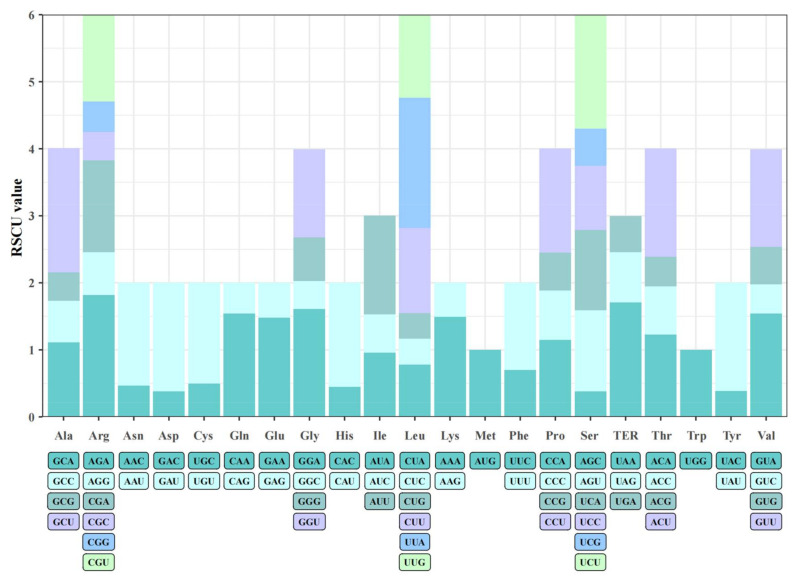
RSCU characteristics of chloroplast genome in *M. zhaojiaoensis*.

The relative synonymous codon usage (RSCU) can represent the relative probability of using synonymous codons and reflect the usage bias of different codons [34]. The total number and relative frequency of all codons were obtained in CodonW software. The results showed that leucine was the most frequent amino acid in the chloroplast coding sequence of *M. zhaojiaoensis*, followed by isoleucine, serine, glycine and arginine. In leucine, codon UUA was widely used. In alanine, the codon GCU was the most frequently used. The arginine bias uses AGA as the codon, and the termination codon bias ends with UAA (Figure 3). There were 30 kinds of codons whose RSCU value was greater than 1, including GCA (Ala), CCA (Pro), UCA (Ser), AGU (Ser), ACA (Thr), UUG (Leu), CUU (Leu), CGU (Arg), UUU (Phe), GGU (Gly), etc. A total of 29 of the 30 codons mentioned above ends with an A/U base, indicating that the third bases of the high-frequency codons are biased toward A/U. In addition, the usage of codons such as AGC, CUG, GAC, UAC and CUC in the cp genome of *M. zhaojiaoensis* was relatively low.

### 3.4. RNA Editing Sites

**Figure 4:**
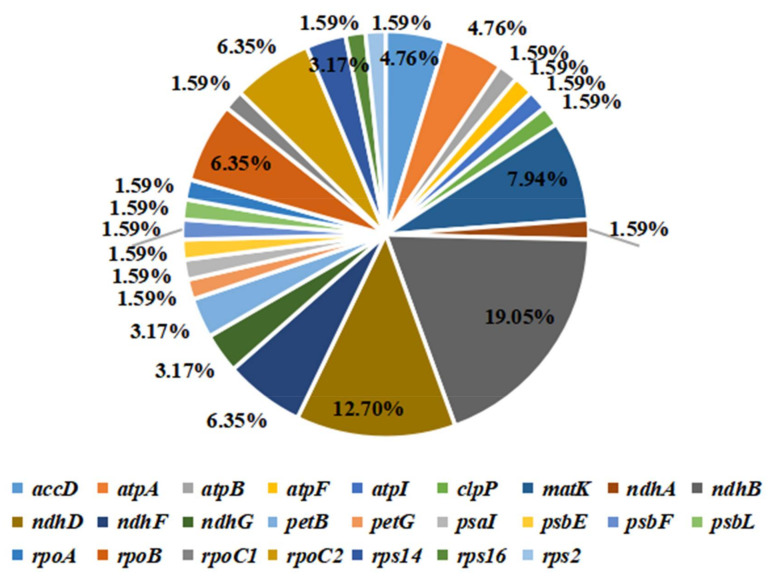
RNA editing sites of CDS in *M. zhaojiaoensis* cp genome. Percentages represent the proportion of potential editing sites in different genes.

RNA editing is a condition in which post-transcribed coding sequence undergoes base conversion that results in changes in amino acids. RNA editing also occurs in plant chloroplast encoded proteins, which can participate in the expression and regulation of genes, and affect and change gene function [35]. In this study, all potential RNA editing sites in the coding sequences of *M. zhaojiaoensis* cp were predicted. The results indicated that a total of 63 RNA editing sites were distributed on 25 genes. These genes mainly included *ndh* (*ndhA*, *ndhB*, *ndhD*, *ndhF*, *ndhG*), *matK*, *rpo* (*rpoA*, *rpoB*, *rpoC1*, *rpoC2*), *accD*, *atp* (*atpA*, *atpB*, *atpF*, *atpI*), *pet* (*petB*, *petG*), *rps* (*rps2*, *rps14*, *rps16*), etc. *ndhB* had the most editing loci with 12, followed by *ndhD* (8), *matK* (5), *ndhF* (4), *rpoB* (4) and *rpoC2* (4) (Figure 4). In terms of the location of RNA editing sites, 48 sites were located at the second base of the codon, accounting for 76.19% of all editing sites (Table S3). In conclusion, in the chloroplast coding genes of *M. zhaojiaoensis*, RNA editing mainly occurred at the second codon. Interestingly, in the CDS of *M. zhaojiaoensis* cp, RNA editing was the most likely to lead to transitions from Serine (S) to Leucine (L), followed by Proline (P) to Leucine (L), and Serine (S) to Phenylalanine (F) (Table S3).

### 3.5. Alignment of Chloroplast Genomes in Malus Species

**Figure 5:**
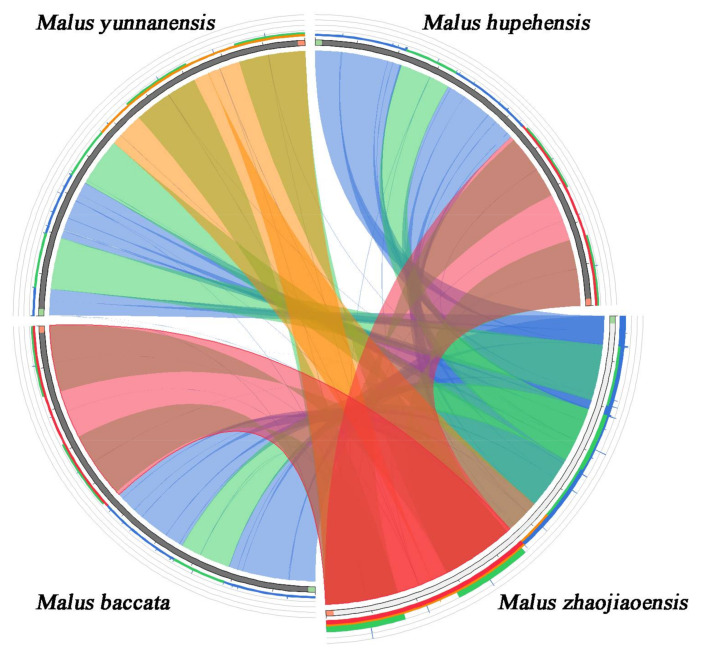
The similarity of the cp genomes of *M. zhaojiaoensis* and three *Malus* species. Different colors represent different comparison areas with different scoring rates. Score/max ratios were colored with blue (≤0.25), green (≤0.50), orange (≤0.75), and red (>0.75).

**Figure 6:**
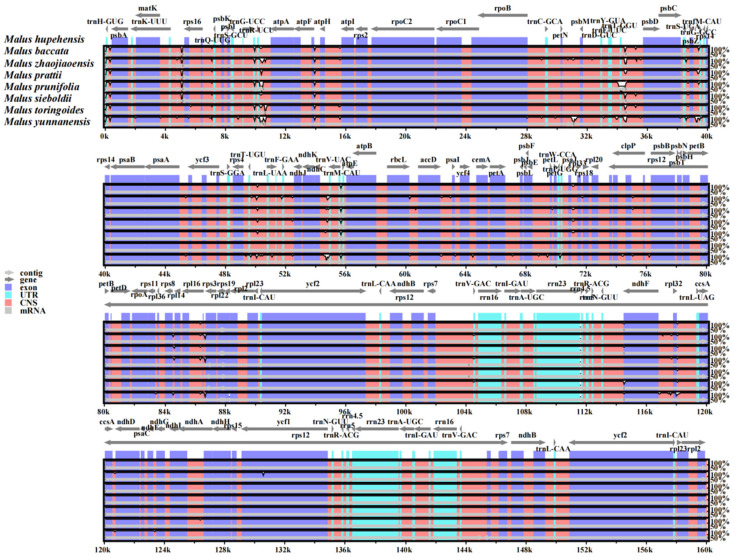
Hotspots of variation in chloroplast genomes of *Malus* species. The abscissa represents the region scope of the cp genomes, and the ordinate shows the similarity ratio of the cp genomes. CNS in the legend refers to non-coding sequences.

In order to compare the sequence similarities and differences of chloroplast genomes of different *Malus* genus, *M. zhaojiaoensis* and three *Malus* species were used for global alignment (Figure 5). The chloroplast sequences of *M. zhaojiaoensis* showed good collinearity with three *Malus* plants. In contrast, the similarity of *M. zhaojiaoensis* to *M. baccata* and *M. hupehensis* was higher, but the similarity to *M. yunnanensis* was slightly lower. To find the variation hotspot regions of cp genomes, eight species of *Malus* (including *M. zhaojiaoensis*) were used for comparative analysis where *M. hupehensis* (MK020147) was a reference. The results showed that most of the differences were located in the LSC region of chloroplast genome, and the other regions were highly similar. It can be clearly observed from Figure 6, that there are large variations in *rps16_trnK-UUU*, *trnG-UCC_atpA*, *atpH_atpF*, *trnT-GGU_psbD*, *psbZ_trnfM-CAU*, *trnV-UAC_ndhC*, *accD_psaI*, *rps3_rpl16*. In general, the hotspots were more distributed in non-coding regions, while gene regions were relatively stable and conserved.

### 3.6. Comparison of Chloroplast Genome Boundaries and Junction Sites

**Figure 7:**
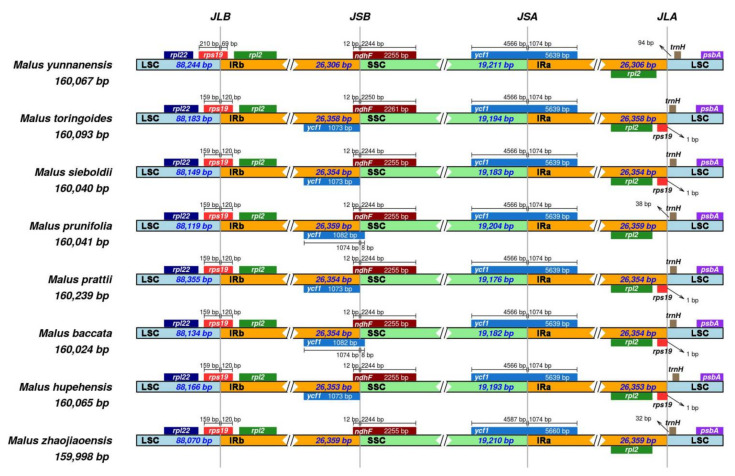
Junction sites and boundary characteristics of eight chloroplast genomes. JLB, JSB, JSA and JLA represent four different junction sites in cp genome boundaries.

JLB (LSC-IRB), JSB (SSC-IRB), JSA (SSC-IRA), JLA (LSC-IRA) are the junction sites on the boundaries of the four regions of the chloroplast genome, which are of great significance in cp genome evolution [36]. In this study, cp genome boundaries of eight *Malus* species were compared in IRscope. As shown in Figure 7, *M. prattii* has the largest cp genome with 160,239 bp, while *M. zhaojiaoensis* cp genome has the smallest sequence length of 159,998 bp. It can be found that the length of LSC region varies from 88,070 to 88,355 bp in eight cp genomes. In contrast, the sequence length of SSC and two IR regions varies little.

For *rps19* gene at the JLB boundary, its location distribution (159 bp in LSC and 120 bp in IRB) was consistent in seven *Malus* species, but the length on the LSC side_*rps19* (210 bp) was longer in *M. yunnanensis*, and sequence length on the IRB side_*rps19* decreased (69 bp). At the JSB site of chloroplast genome of *M. prunifolia* and *M. baccata*, the *ycf1* gene expanded to the SSC region, resulting in the increased length of *ycf1* gene. The *ycf1* gene of IRB/SSC locus did not cross the JSB boundary in *M. toringoides*, *M. sieboldii*, *M. prattii* and *M. hupehensis*. In addition, the displacement of *trnH* gene at JLA locus also occurred in different species (Figure 7). The above results prove that boundary region expansion and contraction play a role in chloroplast genome size and construction.

### 3.7. Phylogenetic Relationships Based on Chloroplast Genomes

**Figure 8:**
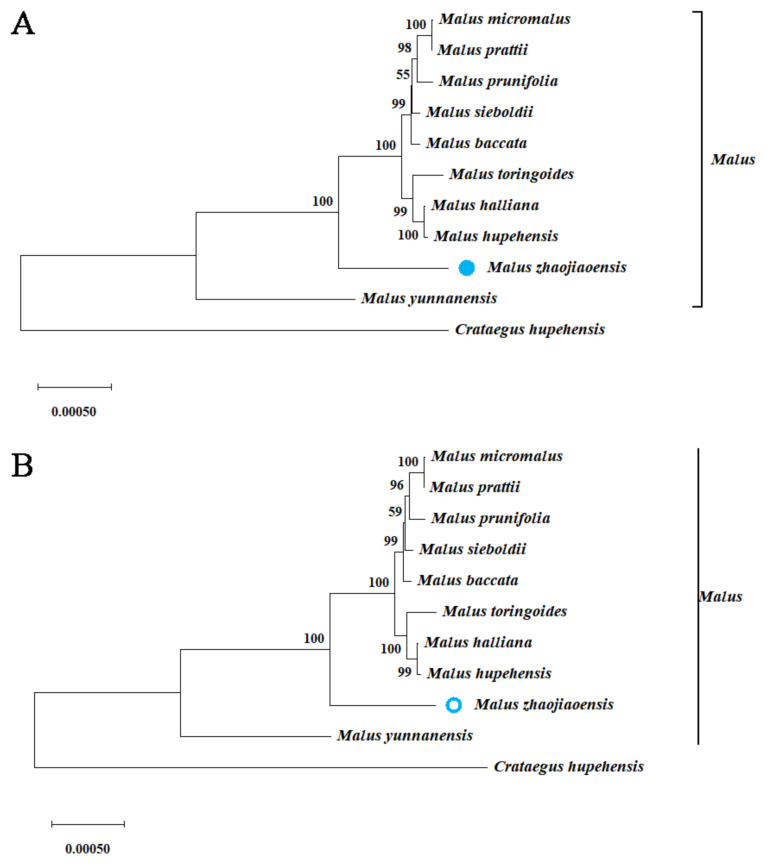
The evolutionary trees of *M. zhaojiaoensis* based on chloroplast sequence. *M. zhaojiaoensis* is highlighted with solid blue circle and hollow blue circle respectively, and the number of the scale reflects the genetic distance. (**A**) Neighbor-joining tree; (**B**) Maximum likelihood tree.

Comparison of chloroplast genome sequences can provide a basis for explaining evolutionary relationships of species. In order to study the evolutionary status of *M. zhaojiaoensis*, chloroplast genomes of 10 species were downloaded from NCBI, including one outgroup (*C. hupehensis*, *Crataegus*). Based on the comparison of 98,748 locations in the dataset, NJ and ML trees for 11 species were constructed. Because the two types of trees were detected by bootstrap method, and their topological structures were very consistent in the results, the evolutionary branches were trustworthy. As can be seen from Figure 8, 11 species were divided into two groups according to genus, and *Malus* plants were grouped into one group. In the *Malus* genus, *M. yunnanensis* was an independent clade due to sequence differences, while *M. zhaojiaoensis* and other eight species form a large clade. The figures in the evolutionary branching shown high support for this result (Figure 8).

**Figure 9:**
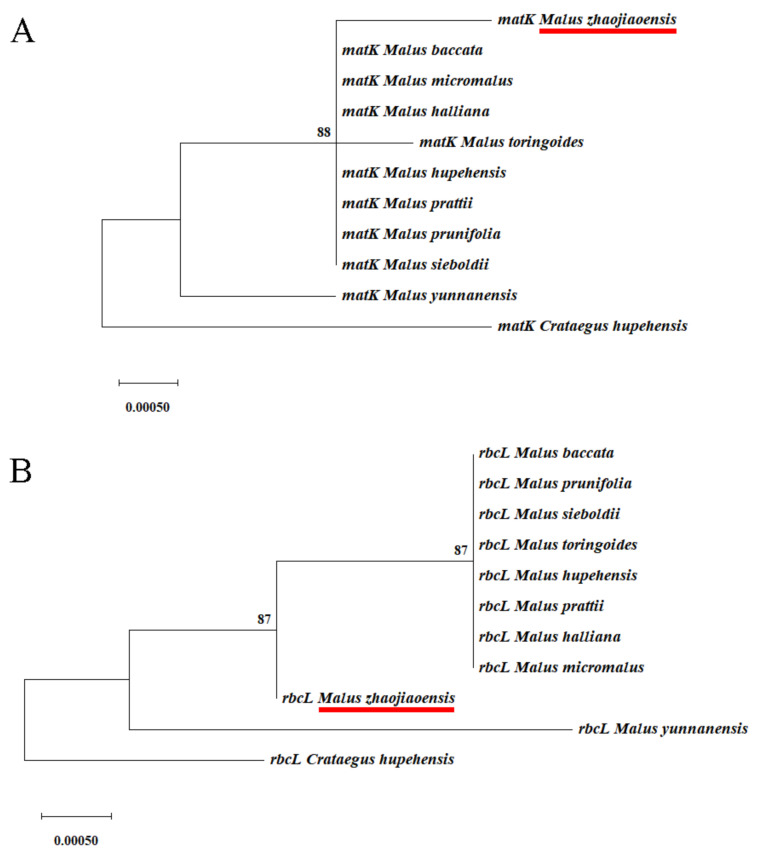
Phylogenetic relationships based on single-copy cp genes *matK* and *rbcL* in 10 *Malus* species. *M. zhaojiaoensis* is highlighted with red underline in (**A**,**B**).

*matK* and *rbcL*, as single-copy genes in chloroplast genome, evolved at a moderate rate and were both suitable for serving as barcodes for plant classification [37]. Hence, evolutionary relationships were characterized based on single-copy chloroplast genes *matK* and *rbcL* from 11 species (Figure 9). The results showed that *M. zhaojiaoensis* and eight species of *Malus* were grouped together, and the clades formed by the two genes were slightly different.

## 4. Discussion

With the development of high-throughput sequencing, more and more chloroplast genomes have been released [38]. Chloroplast genome is different from nuclear genome in that it has the characteristics of maternal inheritance. Because of its small sequence length and moderate base replacement, chloroplast genome has been widely used in the study of genetic variation and phylogeny [39].

In this study, the complete chloroplast genome of *M. zhaojiaoensis* was constructed by whole genome sequencing. Its cp size (159,998 bp) was similar to that of reported *Malus* species, including *M. baccata* (160,024 bp), *M. hupehensis* (160,065 bp), *M. sieboldii* (160,040 bp) and *M. prunifolia* (160,041 bp), etc. This indicates that the length of interspecific chloroplast sequence within *Malus* genus was relatively conserved and the variation was relatively moderate [40]. Further, by annotating the cp sequence of *M. zhaojiaoensis*, it was found that the ring-structured genome consists of four parts, namely LSC, SSC, IRA and IRB. Comparative analysis of different species showed that the LSC regions of the *Malus* cp genomes are highly variable, which contain many hot spots, for example, *trnG-UCC_atpA* and *trnT-GGU_psbD*. The proportion of SSR (75.95%) and INE (60.47%) repeat markers in the LSC region of *M. zhaojiaoensis* was larger than that of the other three regions, which also proved the above inference.

For chloroplast genes, 78 CDS, 29 tRNA, and 4 rRNA were annotated in *M. zhaojiaoensis*. But there were 111 genes in *M. baccata* cp genome, which included 76 protein coding genes, 31 tRNA genes and 4 rRNA genes. In addition, the number of tRNA in chloroplast of *M. hupehensis* was one more than that of *M. zhaojiaoensis*. One of the most important factors contributing to the difference in gene numbers between species may be the expansion and contraction of chloroplast genome boundaries [41]. For example, comparative analysis showed that the *rps19* gene at the JLA junction site was missing in three *Malus* plants (*M. zhaojiaoensis*, *M. prunifolia* and *M. yunnanensis*).

Chloroplast coding sequences were evolutionarily conserved due to low variation. The codons of chloroplast CDS of *M. zhaojiaoensis* were calculated and the results suggested that the high frequency codons were mainly UUA (Leu), GCU (Ala), AGA (Arg) and so on. In *Ocotea aciphylla*, there were 30 high-frequency codons whose RSCU value was greater than 1, including AGA (Arg), GGA (Gly) and UCA (Ser), etc. [42]. In *M. zhaojiaoensis* cp, 29 of the 30 high-frequency codons ended with an A/U base, which reflects the biased use of codons. The similar phenomenon has been found in previous reports [43]. RNA editing was widely used in higher plants and was also an important means of regulating chloroplast gene expression. A total of 63 loci were identified in the coding sequences of *M. zhaojiaoensis* cp and distributed in 25 genes. *ndh* (Subunits of NADH-dehydrogenase) genes had the most potential RNA editing sites (27), followed by *rpo* (DNA dependent RNA polymerase) genes (10 sites). In addition, the predicted results suggested that RNA editing tends to lead to an increase in hydrophobic amino acids (Leucine, Phenylalanine), which is consistent with the study of *Platanthera ussuriensis* [44]. In *Dipterygium glaucum* and *Cleome chrysantha* cp, the amino acids Serine to Leucine were the majority of the conversion in RNA editing [45], which also supports the above speculation.

Chloroplast genome plays an irreplaceable role in species evolution and classification [46]. The information of chloroplast genome can be used to explain the influence of maternal inheritance [47]. The comparison of genetic evolution in *Malus* species has been a long-standing concern of researchers [48]. In this study, based on chloroplast genome and two single copy genes, the evolutionary relationship of *Malus* species was analyzed. The branches and topologies of evolutionary trees were consistent in different methods and datasets. Based on the above results, it can be clearly inferred that *M. yunnanensis* was located in the base group of *Malus* [49], and *M. zhaojiaoensis* formed a large group with 8 species of *Malus*, but was relatively independent in differentiation. In addition, there was further differentiation between *M. toringoides*, *M. halliana* and *M. hupehensis*, while the other five *Malus* species also closely influenced each other. The findings of this paper are consistent with the previous conjecture [6], indicating that *M. zhaojiaoensis* is relatively primitive in evolution.

Due to unique geographical conditions and climate distribution, China is rich in wild plant types and contributes significantly to the world’s biodiversity. *M. zhaojiaoensis* is a wild *Malus* species of China. The exploration of biological classification and genetic relationships of *M. zhaojiaoensis* can speed up the process of conservation and utilization, which provides a good reference for the management and development of wild resources.

## 5. Conclusions

In this study, the complete chloroplast genome of *M. zhaojiaoensis* was sequenced and assembled. Based on the annotation of the cp genome, the structure and sequence characteristics of the genome were analyzed. Prediction of repeat sequence types and RNA editing sites provides potential genetic markers and regulatory loci. Hot spots, boundary features, and evolutionary relationships of *M. zhaojiaoensis* and *Malus* were described by comparative genomics and phylogenetic analysis. The release of chloroplast genome of *M. zhaojiaoensis* can provide valuable support for genetic variation and germplasm identification of *Malus* species. The research in this paper is helpful to the conservation and development of wild *Malus* resources.

## Figures and Tables

**Table 2:** Distribution and number of introns in *M. zhaojiaoensis* cp genome.

Gene	Type	Location	Strand	Start	End	Exon Size	Intron Number
*rps16*	CDS	LSC	Reverse	5247	6378	270	1
*atpF*	CDS	LSC	Reverse	12,444	13,730	555	1
*rpoC1*	CDS	LSC	Reverse	21,865	24,651	2046	1
*ycf3*	CDS	LSC	Reverse	45,423	47,382	507	2
*rps12*	CDS	LSC-IRB	Reverse	73,406	102,714	372	2
*rps12*	CDS	LSC-IRA	Forward	73,406	146,153	372	2
*clpP*	CDS	LSC	Reverse	73,684	75,746	591	2
*petB*	CDS	LSC	Forward	78,684	80,128	648	1
*petD*	CDS	LSC	Forward	80,319	81,525	483	1
*rpl16*	CDS	LSC	Reverse	85,094	86,489	408	1
*rpl2*	CDS	IRB	Reverse	88,261	89,771	825	1
*ndhB*	CDS	IRB	Reverse	98,873	101,074	1533	1
*ndhA*	CDS	SSC	Reverse	124,864	127,080	1092	1
*ndhB*	CDS	IRA	Forward	146,995	149,196	1533	1
*rpl2*	CDS	IRA	Forward	158,298	159,808	825	1
*trnK-UUU*	tRNA	LSC	Reverse	1703	4271	72	1
*trnG-UCC*	tRNA	LSC	Forward	9092	9860	71	1
*trnL-UAA*	tRNA	LSC	Forward	50,581	51,181	85	1
*trnV-UAC*	tRNA	LSC	Reverse	54,758	55,424	75	1
*trnI-GAU*	tRNA	IRB	Forward	106,519	107,538	72	1
*trnA-UGC*	tRNA	IRB	Forward	107,603	108,482	73	1
*trnA-UGC*	tRNA	IRA	Reverse	139,587	140,466	73	1
*trnI-GAU*	tRNA	IRA	Reverse	140,531	141,550	72	1

## Data Availability

All data generated during this study are included in this published article.

## Supplementary Materials

The following supporting information can be downloaded at: https://www.mdpi.com/article/10.3390/genes13040560/s1, Table S1: Type and number of SSRs in *M. zhaojiaoensis* cp genome; Table S2: Type and size of INEs in *M. zhaojiaoensis* cp genome; Table S3: RNA editing sites in *M. zhaojiaoensis* cp genome.
