# Supplementary material for: Identification of the Complete Chloroplast Genome of Malus zhaojiaoensis Jiang and Its Comparison and Evolutionary Analysis with Other Malus Species
Source: Genes (Basel). 2022 Mar 22;13(4):560. doi: 10.3390/genes13040560 (PMC9028542; doi:10.3390/genes13040560)
Supplement: Supplementary file 1 [file genes-13-00560-s001.zip › Table S2.pdf]

Table S2. Type and size of INEs in *M. zhaojiaoensis* cp genome.

INE type	Repeats size	Repeat_1_position	Repeat_2_position	Repeats distance	E-value
P	63	55,834	55,834	-1	1.60E-26
P	44	78,042	78,042	0	2.33E-17
P	48	10,224	10,224	-2	9.23E-16
F	40	62,708	62,727	0	5.96E-15
F	40	102,751	125,440	0	5.96E-15
P	40	125,440	145,277	0	5.96E-15
F	38	39,097	39,124	0	9.53E-14
F	39	46,586	102,753	-1	2.79E-12
P	39	46,586	145,276	-1	2.79E-12
F	38	46,586	125,442	-1	1.09E-11
P	30	8,343	48,047	0	6.24E-09
F	34	54,421	54,455	-2	1.23E-07
F	34	95,430	95,448	-2	1.23E-07
P	34	95,430	152,586	-2	1.23E-07
P	34	95,448	152,604	-2	1.23E-07
F	34	152,586	152,604	-2	1.23E-07
P	31	195	195	-1	1.45E-07
F	33	111,706	111,737	-2	4.64E-07
P	33	111,706	136,298	-2	4.64E-07
P	33	111,737	136,329	-2	4.64E-07
F	33	136,298	136,329	-2	4.64E-07
P	32	54,685	54,685	-2	1.74E-06
R	32	70,856	70,856	-2	1.74E-06
F	31	71,546	71,572	-2	6.53E-06
P	33	13,874	13,874	-3	1.44E-05
P	30	113,964	113,964	-2	2.44E-05
F	30	113,964	134,074	-2	2.44E-05
P	30	134,074	134,074	-2	2.44E-05
F	32	41,597	43,821	-3	5.23E-05
F	32	93,008	93,029	-3	5.23E-05
P	32	93,008	155,007	-3	5.23E-05
P	32	93,029	155,028	-3	5.23E-05
F	32	155,007	155,028	-3	5.23E-05
P	31	199	17,289	-3	1.89E-04
F	31	8,342	38,274	-3	1.89E-04
F	31	10,453	10,493	-3	1.89E-04
P	31	52,216	68,655	-3	1.89E-04
C	30	10,232	34,361	-3	6.85E-04
F	30	10,314	10,336	-3	6.85E-04
P	30	38,275	48,047	-3	6.85E-04
F	30	54,589	54,664	-3	6.85E-04
F	30	54,647	54,671	-3	6.85E-04
F	30	62,699	62,737	-3	6.85E-04

F: forward repeat; R: reverse repeat; C: complement repeat; P: palindromic repeat.
