# Supplementary material for: Identification of the Complete Chloroplast Genome of Malus zhaojiaoensis Jiang and Its Comparison and Evolutionary Analysis with Other Malus Species
Source: Genes (Basel). 2022 Mar 22;13(4):560. doi: 10.3390/genes13040560 (PMC9028542; doi:10.3390/genes13040560)
Supplement: Supplementary file 1 [file genes-13-00560-s001.zip › Table S3.pdf]

Table S3. RNA editing sites in *M. zhaojiaoensis* cp genome.

Gene name	Location of nucleotide	Location of amino acid	RNA editing	Score
ndhB	149	50	TCA (S) => TTA (L)	1
	467	156	CCA (P) => CTA (L)	1
	542	181	ACG (T) => ATG (M)	1
	586	196	CAC (H) => TAC (Y)	1
	611	204	TCA (S) => TTA (L)	0.8
	737	246	CCA (P) => CTA (L)	1
	746	249	TCT (S) => TTT (F)	1
	830	277	TCA (S) => TTA (L)	1
	836	279	TCA (S) => TTA (L)	1
	1,112	371	TCA (S) => TTA (L)	1
	1,255	419	CAT (H) => TAT (Y)	1
	1,481	494	CCA (P) => CTA (L)	1
ndhA	341	114	TCA (S) => TTA (L)	1
ndhG	314	105	ACA (T) => ATA (I)	0.8
	494	165	GCC (A) => GTC (V)	0.8
ndhD	313	105	CGG (R) => TGG (W)	0.8
	383	128	CCA (P) => CTA (L)	1
	599	200	TCA (S) => TTA (L)	1
	674	225	TCA (S) => TTA (L)	1
	878	293	TCA (S) => TTA (L)	1
	887	296	CCC (P) => CTC (L)	1
	1,298	433	TCA (S) => TTA (L)	0.8
	1,405	469	CTT (L) => TTT (F)	0.8
ndhF	290	97	TCG (S) => TTG (L)	1
	392	131	TCC (S) => TTC (F)	1
	1,502	501	ACG (T) => ATG (M)	0.8
	1,876	626	CTT (L) => TTT (F)	1
rpoA	830	277	TCA (S) => TTA (L)	1
petB	418	140	CGG (R) => TGG (W)	1
	611	204	CCA (P) => CTA (L)	1
clpP	559	187	CAT (H) => TAT (Y)	1
petG	13	5	CTT (L) => TTT (F)	0.86
psbE	214	72	CCT (P) => TCT (S)	1
psbF	77	26	TCT (S) => TTT (F)	1
psbL	2	1	ACG (T) => ATG (M)	1
psaI	83	28	TCT (S) => TTT (F)	0.86
accD	64	22	CGG (R) => TGG (W)	1
	854	285	TCG (S) => TTG (L)	0.8
	1,463	488	CCT (P) => CTT (L)	1
atpB	37	13	CCT (P) => TCT (S)	1
rps14	80	27	TCA (S) => TTA (L)	1
	149	50	CCA (P) => CTA (L)	1
rpoB	338	113	TCT (S) => TTT (F)	1
	566	189	TCG (S) => TTG (L)	1
	1,994	665	TCT (S) => TTT (F)	1
	2,420	807	TCA (S) => TTA (L)	0.86
rpoC1	80	27	ACC (T) => ATC (I)	0.86

rpoC2	1,493	498	ACG (T) => ATG (M)	0.86
	1,783	595	CGT (R) => TGT (C)	0.86
	3,062	1,021	TCT (S) => TTT (F)	0.86
	3,740	1,247	TCA (S) => TTA (L)	0.86
rps2	248	83	TCA (S) => TTA (L)	1
atpI	629	210	TCA (S) => TTA (L)	1
atpF	92	31	CCA (P) => CTA (L)	0.86
atpA	773	258	TCA (S) => TTA (L)	1
	914	305	TCA (S) => TTA (L)	1
	1,148	383	TCA (S) => TTA (L)	1
rps16	212	71	TCA (S) => TTA (L)	0.83
matK	532	178	CTC (L) => TTC (F)	0.86
	619	207	CTC (L) => TTC (F)	1
	634	212	CAT (H) => TAT (Y)	1
	923	308	TCT (S) => TTT (F)	0.86
	1,184	395	TCG (S) => TTG (L)	0.86
