# Supplementary material for: Identification of the Complete Chloroplast Genome of Malus zhaojiaoensis Jiang and Its Comparison and Evolutionary Analysis with Other Malus Species
Source: Genes (Basel). 2022 Mar 22;13(4):560. doi: 10.3390/genes13040560 (PMC9028542; doi:10.3390/genes13040560)
Supplement: Supplementary file 1 [file genes-13-00560-s001.zip › Table S1.pdf]

Table S1. Type and number of SSRs in *M. zhaojiaoensis* cp genome.

SSR type	SSR sequence	SSR length	Start	End
c	(T)14agt(A)15	32	196	227
p1	(T)11	11	1,631	1,641
p1	(T)11	11	2,784	2,794
p1	(A)11	11	5,664	5,674
p1	(A)10	10	6,834	6,843
p1	(A)12	12	6,990	7,001
p1	(T)11	11	7,117	7,127
p1	(A)13	13	7,890	7,902
p1	(T)11	11	9,321	9,331
p1	(T)10	10	12,402	12,411
p1	(T)14	14	13,121	13,134
p1	(T)11	11	13,475	13,485
p1	(A)12	12	14,446	14,457
p1	(C)10	10	14,981	14,990
p1	(A)12	12	15,195	15,206
p1	(T)15	15	15,450	15,464
p1	(T)14	14	17,295	17,308
p1	(T)11	11	19,542	19,552
p2	(TA)5	10	20,923	20,932
p1	(T)10	10	27,244	27,253
p1	(T)12	12	27,889	27,900
p1	(A)12	12	28,322	28,333
p1	(A)12	12	28,742	28,753
c	(TA)5ttatatatacgtaattaaatagatttc(TA)5	47	31,139	31,185
p4	(TTTA)3	12	31,804	31,815
p1	(A)14	14	32,141	32,154
p1	(T)19	19	33,077	33,095
c	(TA)5atatagatgcatgatccagcaagcatgcccctttgtaaagt(A)13tg gattcatggtaaaatccttacatgatgca(T)14	109	38,504	38,612
c	(A)11taaaatgaaat(TTTA)3	34	39,222	39,255
c	(T)10gtc(A)10	23	39,552	39,574
p1	(A)10	10	45,191	45,200
p1	(T)10	10	45,720	45,729
p1	(A)10	10	47,550	47,559
p2	(AT)5	10	49,801	49,810
p1	(A)13	13	50,237	50,249
c	(T)10agtatttattttatttagcccacccaataact(A)21	63	52,188	52,250
p1	(T)10	10	53,664	53,673
p2	(TA)5	10	54,692	54,701
p1	(T)10	10	57,764	57,773
p1	(T)20	20	60,495	60,514
p2	(TA)5	10	62,611	62,620
p2	(TA)5	10	62,801	62,810
p2	(TC)5	10	64,631	64,640
c	(T)16atatataggtttattttatcataaccc(T)14	57	66,879	66,935
p1	(G)14	14	68,226	68,239
p1	(T)18	18	68,656	68,673

p1	(A)12	12	68,986	68,997
p2	(AT)5	10	70,057	70,066
p1	(A)16	16	70,907	70,922
p2	(AT)6	12	71,525	71,536
p1	(T)11	11	72,075	72,085
p1	(T)13	13	72,794	72,806
p1	(T)14	14	74,243	74,256
c	(A)12gaatcaatgtgtagatgtagattctagcgctttctttta(T)19	70	74,925	74,994
p2	(AT)5	10	75,935	75,944
p1	(A)10	10	81,711	81,720
c	(T)16acttatttat(TTTA)3	38	83,298	83,335
p1	(T)10	10	84,463	84,472
c	(T)10atcggtttttctttttcaatgcaaaggtataaat(A)14	59	84,950	85,008
p1	(T)19	19	86,285	86,303
p1	(T)10	10	104,086	104,095
p5	(GGCAA)3	15	104,414	104,428
p1	(A)11	11	112,075	112,085
p4	(TTTA)3	12	112,480	112,491
p2	(TA)5	10	113,277	113,286
c	(A)21ttatcttaattaattgtttctgagtcaccggttcttatttcttttcttttgaaa ggggtcggttaat(A)10	98	116,674	116,771
p1	(T)14	14	117,534	117,547
p2	(AT)6	12	118,614	118,625
p1	(T)11	11	123,963	123,973
p2	(AT)5	10	124,792	124,801
p1	(T)10	10	125,876	125,885
p1	(A)10	10	126,431	126,440
p1	(T)10	10	131,803	131,812
p1	(A)16	16	132,434	132,449
p2	(AT)5	10	134,782	134,791
p4	(AATA)3	12	135,576	135,587
p1	(T)11	11	135,984	135,994
p5	(CCTTG)3	15	143,639	143,653
p1	(A)10	10	143,974	143,983

p1 refers to mononucleotide repetition, p2 refers to dinucleotide repetition, c refers to compound repetition.
